# *trans*-Di­chlorido­bis­[(*S*)-(−)-1-(4-methyl­phen­yl)ethyl­amine-κ*N*]palladium(II)

**DOI:** 10.1107/S2414314624000361

**Published:** 2024-01-12

**Authors:** Guadalupe Hernández Téllez, Gloria E. Moreno Morales, Pankaj Sharma, Rodary Gonzalez, Bertin Anzaldo

**Affiliations:** a Lab. Síntesis de Complejos, Fac. Cs. Quím.-BUAP, Ciudad Universitaria, PO Box 72592 Puebla, Mexico; b Instituto de Química Universidad Autónoma de México UNAM, Circuito Exterior Cd. Universitaria, PO Box 04510, Ciudad de México, Mexico; Vienna University of Technology, Austria

**Keywords:** crystal structure, amine, palladium(II) complex, monodentate ligand

## Abstract

The title complex, [PdCl_2_(C_9_H_13_N)_2_], comprises a single mol­ecule in the asymmetric unit. The Pd^II^ atom is tetra­coordinated by two N atoms from two *trans*-aligned organic ligands and two Cl ligands, forming a square-planar metal coordination environment. The distances from the *ortho*-H atoms on the phenyl ring to the central Pd^II^ atom fall within the range 4.70–5.30 Å, precluding any significant intra­molecular Pd⋯H inter­actions.

## Abstract 2

The mol­ecular Pd^II^ title complex exhibits a square-planar coordination about the central metal atom, provided by two *trans*-arranged chlorido ligands and two nitro­gen atoms from the two neutral organic ligands.

## Graphical abstract

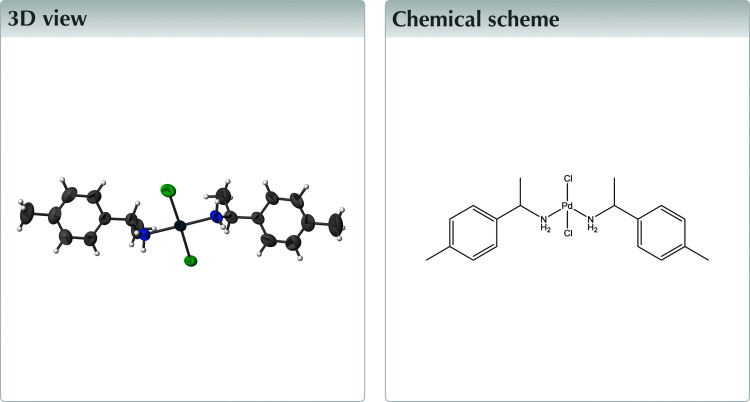

## Structure description

The chemistry of Pd^II^ compounds with diverse ligands represents a rich area within organometallic chemistry, extensively explored in organic synthesis (Hartwig, 1998 ▸; Müller & Beller, 1998 ▸). Pd^II^ compounds also exhibit cytotoxic activity, which makes them inter­esting for certain therapeutic applications. Moreover, Pd^II^ compounds with amine ligands have a central role in catalytic conversions due to the hydrogen bond developed between the amino group and the catalyst. In the presence of excess amine, 16-electron PdCl_2_ *L*_2_ (*L* = amine) adducts, usually existing as a mixture of *cis* and *trans* isomers, emerge as viable starting materials for cyclo­palladations (Ryabov, 1990 ▸; Cattalini & Martelli, 1969 ▸). While monodentate Pd^II^–amine complexes tend to display general instability as reaction inter­mediates, bis­(amine)–Pd^II^ complexes have garnered substantial attention for their involvement as inter­mediates in amination reactions (Widenhoefer & Buchwald, 1996 ▸; Seligson & Trogler, 1991 ▸). In this context, our focus has shifted towards complexes derived from optically pure chiral amines. We present here the mol­ecular and crystal structures of *trans*-di­chlorido bis­[(*S*)-(−)-1-[(4-methyl­phen­yl)ethylamine]­palladium(II).

**Figure 1:**
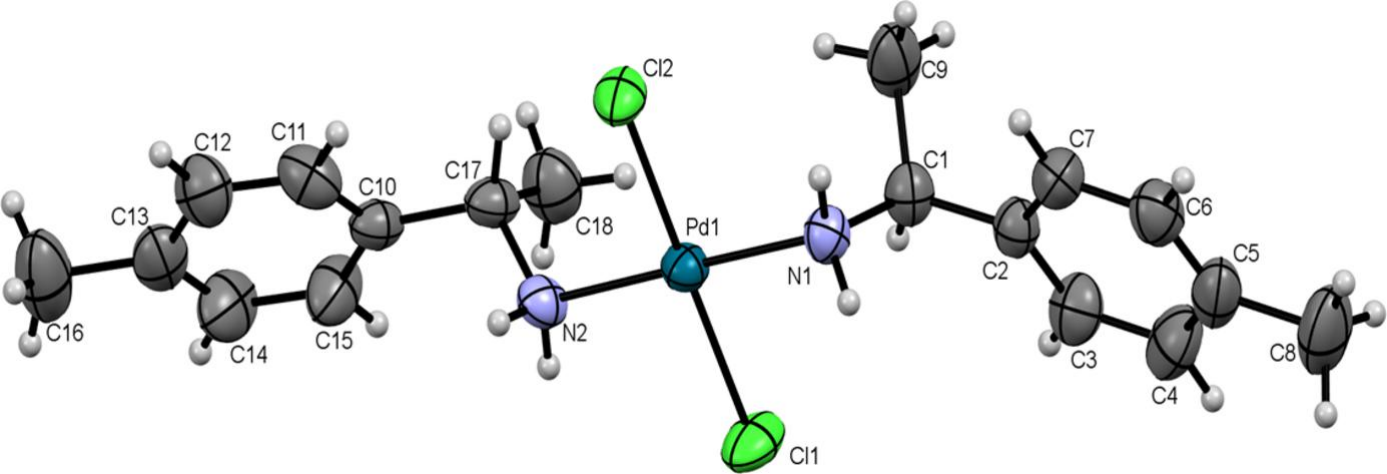
The mol­ecular structure of the title complex with displacement ellipsoids drawn at the 50% probability level.

The asymmetric unit comprises a single mol­ecule, as shown in Fig. 1 ▸. The mol­ecular complex adopts a square-planar metal coordination environment around the central Pd^II^ atom. There are slight distortions from the ideal square-planar geometry, as revealed by a deviation of 0.025 Å of the Pd^II^ atom from the plane defined by atoms Cl2, N2, Cl1, N1. The inter­atomic distances from the central Pd^II^ atom to the ligand atoms are 2.039 (4) Å [Pd1—N1] and 2.053 (4) Å [Pd1—N2]; the average Pd—Cl bond length is 2.298 Å. The pairs of Cl and amine ligands are *trans*-aligned around the central Pd^II^ atom and characterized by a Cl1—Pd1—Cl2 angle of 177.22 (6)° and an N1—Pd1—N2 angle of 179.39 (18)°; the Cl1—Pd1—N1 angle amounts to 88.25 (12)°, with other angles approximately 90°. The *sp* ^3^ hybridization of the N atoms and the C9 and C17 atoms cause the non-planarity of the mol­ecular structure. The amine ligands are arranged differently around the central Pd^II^ atom. The Cl1—Pd1—N1—C1 torsion angle is 73.2 (3)°, compared to 53.5 (3)° for Cl2—Pd1—N2—C17. Both amine ligands exhibit a *gauche* conformation, as revealed by the torsion angle C17—N2—N1—C1 = −55.6 (4)°.

**Figure 2:**
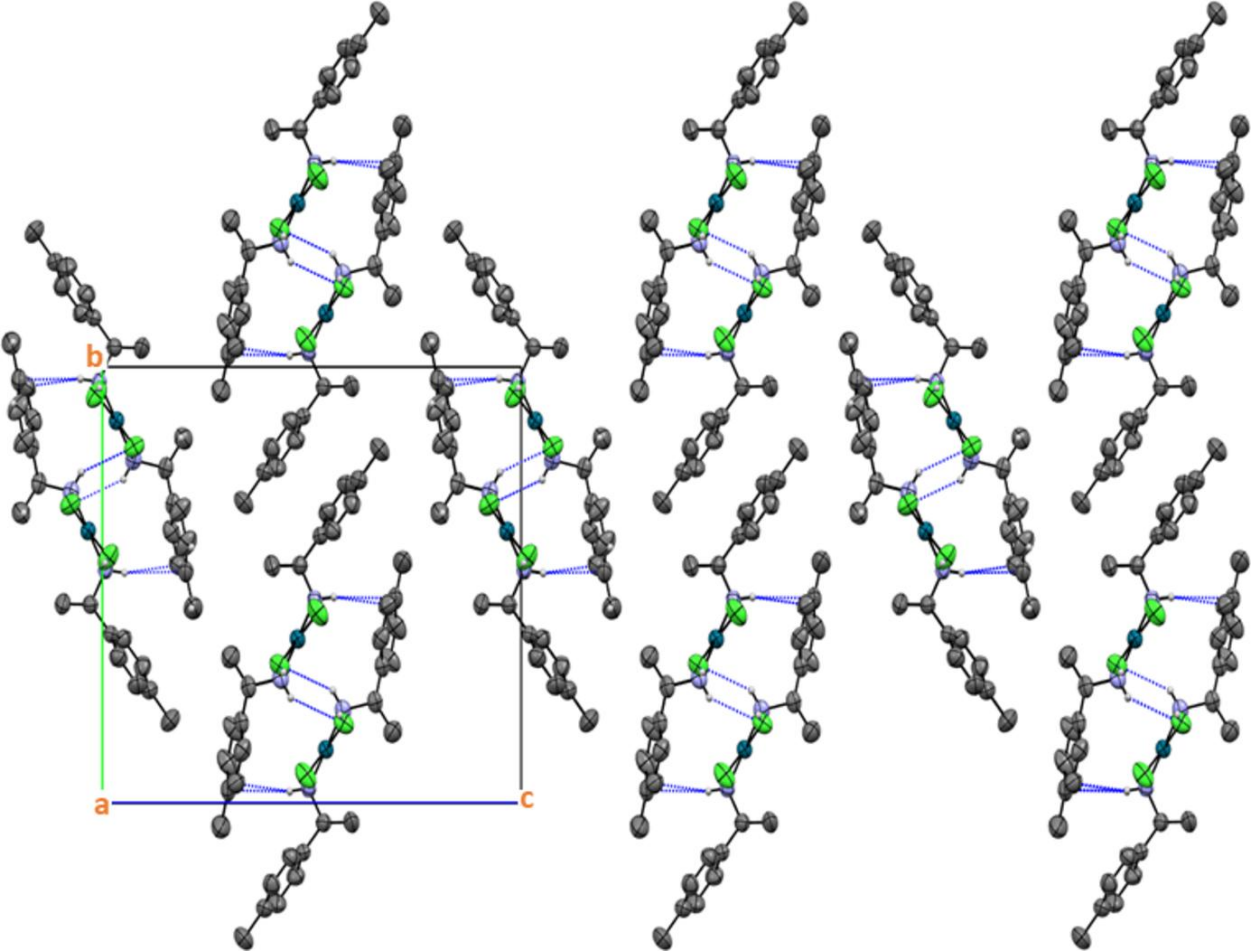
The crystal packing of the title complex in a projection along [100]. The dashed lines indicate inter­molecular hydrogen bonds. All H atoms that are not involved in these inter­actions have been omitted for clarity; displacement ellipsoids are drawn at the 50% probability level.

A view of the crystal packing shows that individual mol­ecules are organized into supra­molecular ribbons defined by C—H⋯Cl and N—H⋯Cl hydrogen bonding inter­actions (Table 1 ▸); the ribbons extend parallel to [100] (Fig. 2 ▸). The cohesion between the ribbons is accomplished mainly by weak van der Waals inter­actions (Steiner, 1996 ▸; Desiraju, 1996 ▸). The Pd⋯Pd separations between neighboring Pd^II^ complexes vary from 5.5027 (5) to 6.5385 (5) Å, indicating that there is no strong inter­action among these metal atoms.

A search of the Cambridge Structural Database (CSD, version 5.42, current as of November 2023; Groom *et al.*, 2016 ▸) yielded thirteen related entries to the title bis­(amine)–Pd^II^ complex: UMIBOH (Sui-Seng & Zargarian, 2003 ▸), UMIBOH01 (Karami *et al.*, 2018 ▸), WOCLEF (Decken *et al.*, 2000 ▸), DUKMAA (Ha, 2020 ▸), BUYCIJ (Al-Jibori *et al.*, 2015 ▸), TUWKEB (Grishin *et al.*, 2003 ▸), YEFNUT (Vazquez *et al.*, 2006 ▸), YEFNUT01 (Sabater *et al.*, 2013 ▸), GAZZAI (Kuz’mina *et al.*, 1987 ▸), GAZZEM (Kuz’mina *et al.*, 1987 ▸), PEWZEY (Karami *et al.*, 2013 ▸), POHKON (Martin *et al.*, 2008 ▸), and CUGGIU (Jones *et al.*, 1984 ▸). In the crystal structure of PEWZEY (*P*2_1_/*c*), mol­ecules are linked by inter­molecular N—H⋯Cl hydrogen bonds into zigzag chains running parallel to the *b* axis. The asymmetric unit of GAZZEM (*P*2_1_) comprises one mol­ecule. In YEFNUT (*C*2), the amine ligands are *trans-*coordinated to a PdCl_2_ core, and arranged in a *gauche* conformation. The asymmetric unit of TUWKEB (*C*2/*c*) comprises one mol­ecule. In DUKMAA (*I*4_1_ *cd*), the complexes and solvent DMSO mol­ecules are linked by N—H⋯O, N—H⋯Cl, C—H⋯Cl and C—H⋯O hydrogen bonds. The crystal structure of UMIBOH crystallizes in space group *P*4_2_/*n* with four independent mol­ecules within the unit cell. The asymmetric unit of GAZZAI (*P*4_3_2_1_2) comprises one mol­ecule. In POHKON (*P*21/*n*), the Pd^II^ atom has a distorted square-planar environment with the ligands occupying a *trans-*configuration with two mol­ecules of dimethyl sulfoxide (DMSO) in the crystal. In the crystal structure of BUYCIJ, a hydrogen bonding inter­action between the water mol­ecule and the metal-bound chlorido ligand is present. CUGGIU comprises a Pd^II^ atom coordinated by the nitro­gen atoms of four benzyl­amine ligands with hydrogen bonding of the N–H_2_ groups with the Cl^−^ ion. The WOCLEF (*P*2_1_/*n*) compound crystallizes with two mol­ecules of DMSO and shows N—H⋯O and C—H⋯Cl hydrogen bonds between the complex and the DMSO mol­ecules.

## Synthesis and crystallization

A solution of bis­(benzo­nitrile)­palladium(II) chloride (0.66 g, 0.17 mmol) in CH_2_Cl_2_ (5 ml) was added to a solution of (*S*)-(+)-[1-(4-methyl­phen­yl)-*N*-(4-biphen­yl)methyl­iden]ethyl­amine (0.100 g, 0.34 mmol) in CH_2_Cl_2_ (10 ml). The solution was stirred for 24 h to give an orange precipitate. The solid was filtered off, dissolved in DMF, and the solution was slowly evaporated. After a few days, orange crystals were collected. Yield 23%.

## Refinement

Crystal data, data collection and structure refinement details are summarized in Table 2 ▸.

## Supplementary Material

Crystal structure: contains datablock(s) I. DOI: 10.1107/S2414314624000361/wm4204sup1.cif

Structure factors: contains datablock(s) I. DOI: 10.1107/S2414314624000361/wm4204Isup2.hkl

CCDC reference: 2310033

Additional supporting information:  crystallographic information; 3D view; checkCIF report

## Figures and Tables

**Table 1:** Hydrogen-bond geometry (Å, °)

*D*—H⋯*A*	*D*—H	H⋯*A*	*D*⋯*A*	*D*—H⋯*A*
C1—H1⋯Cl1	0.98	2.92	3.479 (5)	117
C17—H17⋯Cl2	0.98	2.65	3.323 (5)	126
N1—H1*A*⋯Cl1^i^	0.89	2.71	3.586 (4)	168
N2—H2*A*⋯Cl2^ii^	0.89	2.66	3.524 (4)	165
N2—H2*B*⋯Cl2^iii^	0.89	2.63	3.355 (4)	139

Symmetry codes: (i) 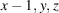 ; (ii) 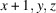 ; (iii) 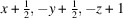 .

**Table 2:** Experimental details

Crystal data	
Chemical formula	[PdCl_2_(C_9_H_13_N)_2_]
*M*_r_	447.71
Crystal system, space group	Orthorhombic, *P*2_1_2_1_2_1_
Temperature (K)	293
*a*, *b*, *c* (Å)	6.5385 (2), 16.7263 (8), 19.0096 (11)
*V* (Å^3^)	2078.99 (17)
*Z*	4
Radiation type	Mo *K*α
μ (mm^−1^)	1.15
Crystal size (mm)	0.58 × 0.38 × 0.14

Data collection	
Diffractometer	Xcalibur, Atlas, Gemini
Absorption correction	Gaussian (*CrysAlis PRO*; Rigaku OD, 2015 ▸)
*T*_min_, *T*_max_	0.722, 0.915
No. of measured, independent and observed [*I* > 2σ(*I*)] reflections	45575, 7907, 5561
*R*_int_	0.061
(sin θ/λ)_max_ (Å^−1^)	0.769

Refinement	
*R*[*F* ^2^ > 2σ(*F* ^2^)], *wR*(*F* ^2^), *S*	0.049, 0.095, 1.05
No. of reflections	7907
No. of parameters	212
H-atom treatment	H-atom parameters constrained
Δρ_max_, Δρ_min_ (e Å^−3^)	0.62, −0.64
Absolute structure	Flack *x* determined using 1800 quotients [(*I* ^+^)−(*I* ^−^)]/[(*I* ^+^)+(*I* ^−^)] (Parsons *et al.*, 2013 ▸)
Absolute structure parameter	−0.032 (18)

Computer programs: *CrysAlis PRO* (Rigaku OD, 2015 ▸), *SHELXD* (Sheldrick, 2008 ▸), *SHELXL* (Sheldrick, 2015 ▸) and *OLEX2* (Dolomanov *et al.*, 2009 ▸).

## full crystallographic data

### Crystal data

**Table:**

[PdCl_2_(C_9_H_13_N)_2_]	*D*_x_ = 1.430 Mg m^−^^3^
*M**_r_* = 447.71	Mo *K*α radiation, λ = 0.71073 Å
Orthorhombic, *P*2_1_2_1_2_1_	Cell parameters from 7901 reflections
*a* = 6.5385 (2) Å	θ = 3.3–27.1°
*b* = 16.7263 (8) Å	µ = 1.15 mm^−^^1^
*c* = 19.0096 (11) Å	*T* = 293 K
*V* = 2078.99 (17) Å^3^	Block, yellow
*Z* = 4	0.58 × 0.38 × 0.14 mm
*F*(000) = 912	

### Data collection

**Table:**

Xcalibur, Atlas, Gemini diffractometer	5561 reflections with *I* > 2σ(*I*)
Detector resolution: 10.5564 pixels mm^-1^	*R*_int_ = 0.061
ω scans	θ_max_ = 33.1°, θ_min_ = 3.3°
Absorption correction: gaussian (CrysAlisPro; Rigaku OD, 2015)	*h* = −10→10
*T*_min_ = 0.722, *T*_max_ = 0.915	*k* = −25→25
45575 measured reflections	*l* = −29→29
7907 independent reflections	

### Refinement

**Table:**

Refinement on *F*^2^	Hydrogen site location: inferred from neighbouring sites
Least-squares matrix: full	H-atom parameters constrained
*R*[*F*^2^ > 2σ(*F*^2^)] = 0.049	*w* = 1/[σ^2^(*F*_o_^2^) + (0.0333*P*)^2^ + 0.5029*P*] where *P* = (*F*_o_^2^ + 2*F*_c_^2^)/3
*wR*(*F*^2^) = 0.095	(Δ/σ)_max_ < 0.001
*S* = 1.05	Δρ_max_ = 0.62 e Å^−^^3^
7907 reflections	Δρ_min_ = −0.64 e Å^−^^3^
212 parameters	Absolute structure: Flack **x determined using 1800 quotients [(*I*^+^)-(*I*^-^)]/[(*I*^+^)+(*I*^-^)] (Parsons *et al.*, 2013)
0 restraints	Absolute structure parameter: −0.032 (18)

### Special details

**Table:**

Geometry. All esds (except the esd in the dihedral angle between two l.s. planes) are estimated using the full covariance matrix. The cell esds are taken into account individually in the estimation of esds in distances, angles and torsion angles; correlations between esds in cell parameters are only used when they are defined by crystal symmetry. An approximate (isotropic) treatment of cell esds is used for estimating esds involving l.s. planes.
Refinement. The hydrogen atoms attached to carbon and nitrogen atoms were positioned with idealized geometry and constrained to ride on their parent atoms, and were refined isotropically using a riding model.

### Fractional atomic coordinates and isotropic or equivalent isotropic displacement parameters (Å^2^)

**Table:**

	*x*	*y*	*z*	*U*_iso_*/*U*_eq_	
Pd1	0.43881 (5)	0.37662 (2)	0.46621 (2)	0.04528 (10)	
Cl1	0.72228 (19)	0.43738 (10)	0.51427 (9)	0.0786 (5)	
Cl2	0.14917 (17)	0.31631 (8)	0.42362 (8)	0.0618 (4)	
C1	0.2846 (8)	0.5452 (3)	0.4715 (3)	0.0574 (12)	
H1	0.427445	0.550708	0.456647	0.069*	
C2	0.2394 (8)	0.6133 (3)	0.5206 (3)	0.0529 (11)	
C3	0.3892 (9)	0.6670 (3)	0.5384 (4)	0.0700 (15)	
H3	0.519174	0.660828	0.519264	0.084*	
C4	0.3531 (12)	0.7299 (4)	0.5837 (4)	0.082 (2)	
H4	0.458570	0.764935	0.594914	0.099*	
C5	0.1610 (12)	0.7412 (3)	0.6126 (3)	0.0743 (17)	
C6	0.0115 (9)	0.6873 (4)	0.5960 (3)	0.0717 (16)	
H6	−0.117513	0.692942	0.616073	0.086*	
C7	0.0475 (9)	0.6243 (3)	0.5499 (3)	0.0658 (13)	
H7	−0.058080	0.589240	0.538736	0.079*	
C8	0.1214 (15)	0.8108 (4)	0.6624 (4)	0.109 (3)	
H8A	−0.020601	0.811728	0.675214	0.163*	
H8B	0.156602	0.860098	0.639512	0.163*	
H8C	0.203350	0.804591	0.703986	0.163*	
C9	0.1548 (13)	0.5439 (4)	0.4054 (3)	0.089 (2)	
H9A	0.194650	0.499443	0.376566	0.133*	
H9B	0.174363	0.592758	0.379829	0.133*	
H9C	0.013344	0.538613	0.418088	0.133*	
C10	0.6754 (7)	0.1854 (3)	0.3317 (3)	0.0538 (12)	
C11	0.5455 (8)	0.1214 (4)	0.3346 (3)	0.0696 (14)	
H11	0.410391	0.129536	0.348156	0.084*	
C12	0.6089 (12)	0.0453 (4)	0.3181 (4)	0.079 (2)	
H12	0.515250	0.003482	0.318976	0.094*	
C13	0.8098 (12)	0.0302 (4)	0.3004 (3)	0.0724 (17)	
C14	0.9381 (11)	0.0941 (4)	0.2986 (4)	0.086 (2)	
H14	1.074562	0.085587	0.287024	0.103*	
C15	0.8753 (8)	0.1713 (4)	0.3133 (4)	0.0789 (19)	
H15	0.967897	0.213352	0.310781	0.095*	
C16	0.8851 (15)	−0.0534 (4)	0.2830 (4)	0.111 (3)	
H16A	0.825566	−0.090992	0.315120	0.166*	
H16B	1.031357	−0.055076	0.286979	0.166*	
H16C	0.845891	−0.066826	0.235748	0.166*	
C17	0.5914 (7)	0.2677 (3)	0.3488 (3)	0.0563 (12)	
H17	0.444373	0.266492	0.338874	0.068*	
C18	0.6817 (12)	0.3357 (4)	0.3063 (4)	0.0852 (19)	
H18A	0.624801	0.385508	0.322108	0.128*	
H18B	0.649956	0.328210	0.257422	0.128*	
H18C	0.827385	0.336610	0.312394	0.128*	
N1	0.2654 (6)	0.4663 (2)	0.5077 (2)	0.0496 (9)	
H1A	0.134790	0.451351	0.506492	0.060*	
H1B	0.299856	0.472545	0.552656	0.060*	
N2	0.6156 (6)	0.2865 (2)	0.4253 (2)	0.0532 (10)	
H2A	0.746127	0.298909	0.432873	0.064*	
H2B	0.589351	0.242141	0.449531	0.064*	

### Atomic displacement parameters (Å^2^)

**Table:**

	*U*^11^	*U*^22^	*U*^33^	*U*^12^	*U*^13^	*U*^23^
Pd1	0.03448 (13)	0.05194 (17)	0.04942 (17)	0.00021 (15)	−0.00095 (15)	−0.00264 (18)
Cl1	0.0396 (6)	0.1033 (11)	0.0927 (12)	−0.0090 (7)	−0.0076 (6)	−0.0358 (9)
Cl2	0.0343 (5)	0.0663 (8)	0.0849 (10)	−0.0047 (5)	−0.0015 (6)	−0.0168 (7)
C1	0.065 (3)	0.053 (3)	0.054 (3)	0.001 (2)	0.008 (3)	0.001 (3)
C2	0.062 (3)	0.046 (3)	0.051 (3)	−0.001 (2)	0.003 (2)	0.001 (2)
C3	0.075 (4)	0.061 (3)	0.074 (4)	−0.011 (3)	0.010 (3)	0.003 (3)
C4	0.107 (5)	0.061 (4)	0.080 (5)	−0.023 (4)	−0.002 (4)	−0.005 (3)
C5	0.110 (5)	0.053 (3)	0.060 (4)	0.004 (3)	−0.004 (4)	−0.003 (3)
C6	0.074 (4)	0.076 (4)	0.064 (4)	0.014 (3)	0.004 (3)	−0.005 (3)
C7	0.070 (3)	0.060 (3)	0.067 (3)	0.001 (3)	−0.009 (3)	−0.007 (3)
C8	0.169 (9)	0.079 (4)	0.079 (5)	0.006 (5)	0.005 (5)	−0.027 (4)
C9	0.139 (6)	0.073 (4)	0.054 (4)	0.022 (4)	−0.004 (4)	−0.007 (3)
C10	0.042 (2)	0.073 (3)	0.046 (3)	0.005 (2)	−0.002 (2)	−0.006 (3)
C11	0.055 (3)	0.084 (4)	0.070 (3)	0.004 (4)	0.008 (3)	0.017 (3)
C12	0.088 (5)	0.067 (4)	0.081 (5)	−0.002 (3)	0.004 (4)	0.013 (3)
C13	0.096 (5)	0.075 (4)	0.046 (3)	0.013 (4)	0.003 (3)	−0.002 (3)
C14	0.055 (3)	0.108 (5)	0.095 (5)	0.015 (4)	0.011 (4)	−0.035 (4)
C15	0.048 (3)	0.091 (4)	0.098 (5)	0.000 (3)	0.011 (3)	−0.036 (4)
C16	0.168 (9)	0.085 (5)	0.079 (5)	0.036 (6)	0.016 (5)	−0.001 (4)
C17	0.042 (3)	0.075 (3)	0.051 (3)	0.007 (2)	−0.005 (2)	−0.006 (3)
C18	0.101 (5)	0.080 (4)	0.075 (5)	0.009 (4)	0.005 (4)	0.008 (4)
N1	0.049 (2)	0.051 (2)	0.049 (2)	0.0013 (17)	0.0052 (18)	−0.0033 (18)
N2	0.0399 (19)	0.065 (2)	0.055 (3)	0.0106 (18)	0.0000 (17)	−0.005 (2)

### Geometric parameters (Å, º)

**Table:**

Pd1—Cl1	2.3028 (13)	C10—C11	1.368 (7)
Pd1—Cl2	2.2933 (12)	C10—C15	1.373 (7)
Pd1—N1	2.039 (4)	C10—C17	1.517 (7)
Pd1—N2	2.053 (4)	C11—H11	0.9300
C1—H1	0.9800	C11—C12	1.374 (8)
C1—C2	1.502 (7)	C12—H12	0.9300
C1—C9	1.516 (9)	C12—C13	1.379 (10)
C1—N1	1.494 (6)	C13—C14	1.360 (9)
C2—C3	1.371 (7)	C13—C16	1.518 (9)
C2—C7	1.385 (7)	C14—H14	0.9300
C3—H3	0.9300	C14—C15	1.382 (8)
C3—C4	1.381 (8)	C15—H15	0.9300
C4—H4	0.9300	C16—H16A	0.9600
C4—C5	1.383 (10)	C16—H16B	0.9600
C5—C6	1.366 (9)	C16—H16C	0.9600
C5—C8	1.524 (8)	C17—H17	0.9800
C6—H6	0.9300	C17—C18	1.515 (8)
C6—C7	1.391 (8)	C17—N2	1.496 (7)
C7—H7	0.9300	C18—H18A	0.9600
C8—H8A	0.9600	C18—H18B	0.9600
C8—H8B	0.9600	C18—H18C	0.9600
C8—H8C	0.9600	N1—H1A	0.8900
C9—H9A	0.9600	N1—H1B	0.8900
C9—H9B	0.9600	N2—H2A	0.8900
C9—H9C	0.9600	N2—H2B	0.8900
			
Cl2—Pd1—Cl1	177.22 (6)	C10—C11—H11	119.1
N1—Pd1—Cl1	88.25 (12)	C10—C11—C12	121.9 (6)
N1—Pd1—Cl2	90.05 (12)	C12—C11—H11	119.1
N1—Pd1—N2	179.39 (18)	C11—C12—H12	119.6
N2—Pd1—Cl1	91.21 (12)	C11—C12—C13	120.9 (7)
N2—Pd1—Cl2	90.48 (12)	C13—C12—H12	119.6
C2—C1—H1	107.2	C12—C13—C16	122.1 (7)
C2—C1—C9	114.6 (4)	C14—C13—C12	116.7 (6)
C9—C1—H1	107.2	C14—C13—C16	121.2 (7)
N1—C1—H1	107.2	C13—C14—H14	118.5
N1—C1—C2	111.5 (4)	C13—C14—C15	123.1 (6)
N1—C1—C9	108.8 (5)	C15—C14—H14	118.5
C3—C2—C1	120.6 (5)	C10—C15—C14	119.7 (6)
C3—C2—C7	117.5 (5)	C10—C15—H15	120.2
C7—C2—C1	122.0 (5)	C14—C15—H15	120.2
C2—C3—H3	119.0	C13—C16—H16A	109.5
C2—C3—C4	122.1 (6)	C13—C16—H16B	109.5
C4—C3—H3	119.0	C13—C16—H16C	109.5
C3—C4—H4	119.8	H16A—C16—H16B	109.5
C3—C4—C5	120.5 (6)	H16A—C16—H16C	109.5
C5—C4—H4	119.8	H16B—C16—H16C	109.5
C4—C5—C8	120.3 (7)	C10—C17—H17	107.2
C6—C5—C4	117.9 (6)	C18—C17—C10	115.2 (5)
C6—C5—C8	121.7 (7)	C18—C17—H17	107.2
C5—C6—H6	119.2	N2—C17—C10	111.1 (4)
C5—C6—C7	121.6 (6)	N2—C17—H17	107.2
C7—C6—H6	119.2	N2—C17—C18	108.6 (5)
C2—C7—C6	120.5 (5)	C17—C18—H18A	109.5
C2—C7—H7	119.8	C17—C18—H18B	109.5
C6—C7—H7	119.8	C17—C18—H18C	109.5
C5—C8—H8A	109.5	H18A—C18—H18B	109.5
C5—C8—H8B	109.5	H18A—C18—H18C	109.5
C5—C8—H8C	109.5	H18B—C18—H18C	109.5
H8A—C8—H8B	109.5	Pd1—N1—H1A	108.5
H8A—C8—H8C	109.5	Pd1—N1—H1B	108.5
H8B—C8—H8C	109.5	C1—N1—Pd1	115.2 (3)
C1—C9—H9A	109.5	C1—N1—H1A	108.5
C1—C9—H9B	109.5	C1—N1—H1B	108.5
C1—C9—H9C	109.5	H1A—N1—H1B	107.5
H9A—C9—H9B	109.5	Pd1—N2—H2A	107.9
H9A—C9—H9C	109.5	Pd1—N2—H2B	107.9
H9B—C9—H9C	109.5	C17—N2—Pd1	117.6 (3)
C11—C10—C15	117.8 (6)	C17—N2—H2A	107.9
C11—C10—C17	118.5 (5)	C17—N2—H2B	107.9
C15—C10—C17	123.7 (5)	H2A—N2—H2B	107.2
			
C1—C2—C3—C4	−179.6 (6)	C11—C10—C15—C14	0.0 (10)
C1—C2—C7—C6	179.1 (5)	C11—C10—C17—C18	−144.6 (6)
C2—C1—N1—Pd1	−153.2 (3)	C11—C10—C17—N2	91.3 (6)
C2—C3—C4—C5	−0.5 (10)	C11—C12—C13—C14	−1.4 (11)
C3—C2—C7—C6	−0.6 (8)	C11—C12—C13—C16	179.1 (6)
C3—C4—C5—C6	1.4 (11)	C12—C13—C14—C15	−0.3 (11)
C3—C4—C5—C8	−179.8 (6)	C13—C14—C15—C10	1.0 (12)
C4—C5—C6—C7	−1.9 (10)	C15—C10—C11—C12	−1.7 (9)
C5—C6—C7—C2	1.5 (9)	C15—C10—C17—C18	35.4 (8)
C7—C2—C3—C4	0.1 (9)	C15—C10—C17—N2	−88.7 (7)
C8—C5—C6—C7	179.3 (6)	C16—C13—C14—C15	179.2 (7)
C9—C1—C2—C3	−120.9 (6)	C17—C10—C11—C12	178.3 (6)
C9—C1—C2—C7	59.5 (7)	C17—C10—C15—C14	180.0 (6)
C9—C1—N1—Pd1	79.5 (5)	C18—C17—N2—Pd1	70.7 (5)
C10—C11—C12—C13	2.4 (10)	N1—C1—C2—C3	115.0 (5)
C10—C17—N2—Pd1	−161.6 (3)	N1—C1—C2—C7	−64.6 (6)

### Hydrogen-bond geometry (Å, º)

**Table:**

*D*—H···*A*	*D*—H	H···*A*	*D*···*A*	*D*—H···*A*
C1—H1···Cl1	0.98	2.92	3.479 (5)	117
C17—H17···Cl2	0.98	2.65	3.323 (5)	126
N1—H1*A*···Cl1^i^	0.89	2.71	3.586 (4)	168
N2—H2*A*···Cl2^ii^	0.89	2.66	3.524 (4)	165
N2—H2*B*···Cl2^iii^	0.89	2.63	3.355 (4)	139

Symmetry codes: (i) *x*−1, *y*, *z*; (ii) *x*+1, *y*, *z*; (iii) *x*+1/2, −*y*+1/2, −*z*+1.
